# Imaging of Joint and Soft Tissue Involvement in Systemic Lupus Erythematosus

**DOI:** 10.1007/s11926-021-01040-8

**Published:** 2021-07-16

**Authors:** Andrea Di Matteo, Gianluca Smerilli, Edoardo Cipolletta, Fausto Salaffi, Rossella De Angelis, Marco Di Carlo, Emilio Filippucci, Walter Grassi

**Affiliations:** Polytechnic University of Marche, Rheumatology Unit, Department of Clinical and Molecular Sciences, “Carlo Urbani” Hospital, Via Aldo Moro 25, 60035 Jesi, Ancona, Italy

**Keywords:** Systemic lupus erythematosus, Musculoskeletal involvement, Imaging, ultrasound (US), Magnetic resonance imaging (MRI), Conventional radiography (CR), Computed tomography (CT)

## Abstract

**Purpose of Review:**

To highlight the potential uses and applications of imaging in the assessment of the most common and relevant musculoskeletal (MSK) manifestations in systemic lupus erythematosus (SLE).

**Recent Findings:**

Ultrasound (US) and magnetic resonance imaging (MRI) are accurate and sensitive in the assessment of inflammation and structural damage at the joint and soft tissue structures in patients with SLE. The US is particularly helpful for the detection of joint and/or tendon inflammation in patients with arthralgia but without clinical synovitis, and for the early identification of bone erosions. MRI plays a key role in the early diagnosis of osteonecrosis and in the assessment of muscle involvement (i.e., myositis and myopathy). Conventional radiography (CR) remains the traditional gold standard for the evaluation of structural damage in patients with joint involvement, and for the study of bone pathology. The diagnostic value of CR is affected by the poor sensitivity in demonstrating early structural changes at joint and soft tissue level. Computed tomography allows a detailed evaluation of bone damage. However, the inability to distinguish different soft tissues and the need for ionizing radiation limit its use to selected clinical circumstances. Nuclear imaging techniques are valuable resources in patients with suspected bone infection (i.e., osteomyelitis), especially when MRI is contraindicated. Finally, dual energy X-ray absorptiometry represents the imaging mainstay for the assessment and monitoring of bone status in patients with or at-risk of osteoporosis.

**Summary:**

Imaging provides relevant and valuable information in the assessment of MSK involvement in SLE.

## Introduction

Systemic lupus erythematosus (SLE) is an autoimmune disease which is characterized by a huge variability of clinical manifestations [1]. The involvement of the musculoskeletal (MSK) system is extremely frequent, being reported in up to 90% of SLE patients [2].

The spectrum of MSK manifestations in SLE is wide and heterogenous. SLE can affect the joints, tendons, muscles, and bones [3]. Articular manifestations range from mild and self-limiting arthralgia to persistent arthritis which can be deforming [i.e., Jaccoud’s arthropathy (JA)] and/or erosive (i.e., ‘Rhupus’) in a small proportion of patients [4]. Tendon inflammation, such as tenosynovitis, is frequently observed. Tendon rupture is a rare but potentially disabling complication of the disease [5]. Recent ultrasound (US) studies have shown that also nonsynovial structures, such as entheses and tendons without synovial sheath, may be involved in SLE [6••, 7, 8]. While diffuse myalgia is reported by up to 50% of SLE patients, overt inflammatory myositis is rarely observed (around 10% of patients) [9]. Osteonecrosis and osteoporosis are important causes of morbidity in SLE [10]. Other possible MSK pathological conditions detectable in SLE include osteomyelitis and septic arthritis.

Imaging has a key role in the diagnosis and characterization of MSK manifestations in SLE patients. US has proven to be sensitive and accurate for the identification of both inflammation and structural damage at joint, tendon, and entheseal level, emerging as the potential first-line imaging technique for the assessment of SLE patients with joint symptoms [11••, 12]. Some studies have also demonstrated the value of magnetic resonance imaging (MRI) in the detection of joint and tendon pathology in SLE patients [13]. The use of MRI is perhaps limited by the traditional concept of joint involvement in SLE being a ‘mild’ condition, as regards long-term outcome and clinical course, other than by feasibility aspects, such as availability, cost, and patient tolerance. Indeed, MRI has a crucial role in the early diagnosis of osteonecrosis, where the sensitivity of conventional radiography (CR) is often low [14], and in the assessment of muscle pathology (i.e., myositis and myopathy) [15]. CR is routinely used for the evaluation of joint damage (i.e., bone erosions, joint space narrowing, and malalignment) in patients with SLE arthropathy. The poor sensitivity in demonstrating early structural changes at joint and soft tissue level represents the main limitation of CR [16]. Computed tomography (CT) is accurate for the detection of bone pathology but its inability to distinguish different soft tissues and the need for ionizing radiation confine its use to selected clinical scenarios. Nuclear imaging (e.g., bone scintigraphy) is particularly helpful in the assessment of bone infection, especially when MRI is contraindicated, or in multifocal osteonecrosis. Dual energy X-ray absorptiometry (DEXA) remains the imaging cornerstone of osteoporosis screening and diagnosis.

In this review, we provide an overview of the studies which highlighted the potential uses and applications of imaging in the assessment of the most common and relevant SLE MSK manifestations.

## SLE Arthropathy

### Inflammatory Arthralgia or Inflammatory Arthritis?

Joint involvement has been long regarded as a minor and mild clinical manifestation in SLE. However, in recent years, several studies have demonstrated the considerable impact of MSK manifestations on the quality of life, functionality, and ability to work in patients with SLE [17–21]. Indeed, these studies have changed the traditional view of ‘SLE-arthritis’, which is now acknowledged as a major feature of the disease.

Inflammatory arthralgia, without clinical signs of joint or tendon inflammation, is commonly observed. Frank arthritis, which can be either fluctuating or persistent, is usually polyarticular and symmetric [22]. Traditionally, three main subsets of SLE arthropathy have been recognized: (1) non-deforming non-erosive (NDNE) arthritis, which is the most common type of SLE arthropathy; (2) deforming arthropathy, also called JA, which is observed in up to 15% of SLE patients and (3) erosive arthropathy (i.e., ‘Rhupus’), which affects the minority (around 5%) of SLE patients [23].

In recent years, several studies have highlighted the value of imaging, namely, US and MRI, in the assessment of articular and peri-articular soft tissue involvement in patients with SLE. At joint and tendon level, US and MRI have shown a wide spectrum of pathological abnormalities indicating inflammation (e.g., synovitis, tenosynovitis, enthesitis, capsular swelling, and bone marrow edema) and/or structural damage (e.g., bone erosions, cartilage thinning, and tendon damage) [6, 13, 24]. An important aspect that has emerged from the US studies evaluating MSK involvement in SLE patients is the remarkable prevalence of joint and/or tendon inflammation in those patients with arthralgia but without clinical signs of synovitis and/or tenosynovitis.

In a study by Torrente-Segarra et al., the presence of US inflammation [i.e., synovial hypertrophy and power Doppler (PD) signal or PD signal only] was investigated in 28 SLE patients with hands and wrists arthralgia but no current or previous arthritis [25]. The scanning protocol included the wrist joints, all metacarpophalangeal (MCP) and proximal interphalangeal (PIP) joints, the flexor tendons at the wrists and all finger extensor tendons. In this study, extensor tendons tenosynovitis and ‘active’ wrist synovitis were found in 39.2% and 14.2% of patients, respectively.

In another study by Gabba et al., US findings of inflammation were found in 20 out of 26 (76.9%) SLE patients with arthralgia but without clinical arthritis [26]. Synovial effusion was the most prevalent US finding, being detected in 50% and 34.6% of patients respectively at tendon and joint level. In addition, almost a quarter of patients had evidence of intra-articular PD signal. The authors of this paper suggest that SLE patients without clinical arthritis but with arthralgia and US inflammation should be regarded as having MSK ‘active’ disease and, as such, should be treated with low-dose prednisone and/or antimalarial drugs.

Similar results were also reported by Dreyer et al., who compared the US findings in SLE patients with arthralgia vs SLE patients without MSK symptoms [27]. In this study, the prevalence of ‘active’ inflammation in the wrists and MCP joints was significantly higher in patients with arthralgia (81%) in comparison with the asymptomatic group (18%, *p*=0.0005). Finally, in a UK study including 122 SLE patients, of whom 88 had inflammatory MSK symptoms, subclinical inflammation on US (without clinically swollen joints) was observed in almost a third of patients [28].

The detection of US joint and/or tendon inflammation in SLE patients with MSK symptoms (i.e., inflammatory arthralgia), but without clinical arthritis, raises potential implications for the management of these patients. To date, patient treatment choice (including ‘treat to target’ protocols) or inclusion in clinical trials are based on clinical disease activity instruments, such as the Systemic Lupus Erythematosus Disease Activity Index 2000 (SLEDAI-2K) or the British Isles Lupus Assessment Group (BILAG)-MSK index, which are all heavily weighted by the presence of synovitis [29, 30]. Consequently, MSK ‘active’ disease may be missed in a considerable proportion of SLE patients without clinical synovitis but with MSK symptoms and inflammation on US. However, additional evidence on the long-term outcome of the US findings is needed before suggesting a more aggressive therapeutic approach in all patients showing US synovitis (or tenosynovitis) but no joint (or tendon) inflammation on physical examination. With this regard, interesting new data come from a very recent multicentric study, in which the presence of US synovitis and/or tenosynovitis was associated with a better response to therapy (i.e., intramuscular 120 mg methylprednisolone) in 133 SLE patients with inflammatory joint pain [31].

### Joint Structural Damage

Except for ‘Rhupus’ [which is by some considered an overlap syndrome between rheumatoid arthritis (RA) and SLE, while by others a distinct erosive subset of SLE arthropathy with a strong association with anti-citrullinated protein antibodies (ACPA) antibodies], joint involvement in SLE is traditionally regarded as nonerosive [32, 33]. Indeed, this traditional concept has been recently questioned by several imaging studies, which have showed a high prevalence of joint structural damage (i.e., bone erosions) in SLE arthropathy-subsets which are traditionally regarded as nonerosive, such as NDNE arthritis and JA [34].

JA is a chronic arthropathy which is characterized by RA-like deformities of the hands and feet (i.e., swan neck deformity, boutonniere deformity, thumb subluxation, MCP joints ulnar deviation, and hallux valgus). The main differences between JA and the articular involvement which is observed in RA are the reducibility of the deformities, at least in the early stages of the disease, and the lack of erosions on CR [35, 36].

We will highlight some of the MRI and US studies that have revealed a high burden of erosive changes in patients with JA. In a study by Ostendorf et al., MRI bone erosions were found in 8 out of 14 (57.1%) patients with SLE, active arthritis, and JA [37]. Bone erosions were found in ≥2 joints in 6 out of 14 (42.9%) patients, mainly at the 2nd and 3rd MCP joints, 2nd and 3rd PIP joints, and at the carpal joints. Interestingly, bone erosions were detected by MRI in almost half of the patients with normal CR (i.e., without bone erosions). In addition, while bone erosions were observed on CR only in a third of patients with JA-related deformities, bony lesions (cysts or erosions) were detected by MRI in more than two-third of patients. In another study, Ball et al. explored the MRI pathological changes at the hands and wrists in 34 SLE patients with arthralgia with or without clinical evidence of joint swelling or deformity; in this study, wrists and MCP joints bone erosions were respectively found in 93% and 61% of patients [38].

A high prevalence of bone erosions in patients with JA has also been documented in some US studies. Ceccarelli et al. found bone erosions in 10 out of 17 (58.8%) patients with JA (i.e., JA index ≥5) [39]. Piga et al. evaluated the accuracy of US in the detection of bone erosions in the MCP joints and wrists of patients with different SLE arthropathy-subsets (i.e., NDNE arthritis, JA, and ‘Rhupus’), using CT as the imaging gold-standard [40]. US detected bone erosions were seen in 5 out of 10 (50.0%) JA patients, mostly at the 2nd MCP joint and wrist joint; CT-detected bone erosions were observed in 8 out of 10 JA patients (80.0%). It should be noted that other than in patients with JA, an unexpectedly high (albeit largely variable in the different studies) prevalence of erosive damage has also been reported in patients with NDNE arthritis, both by US and MRI studies [12, 41, 42].

**Fig. 1.**
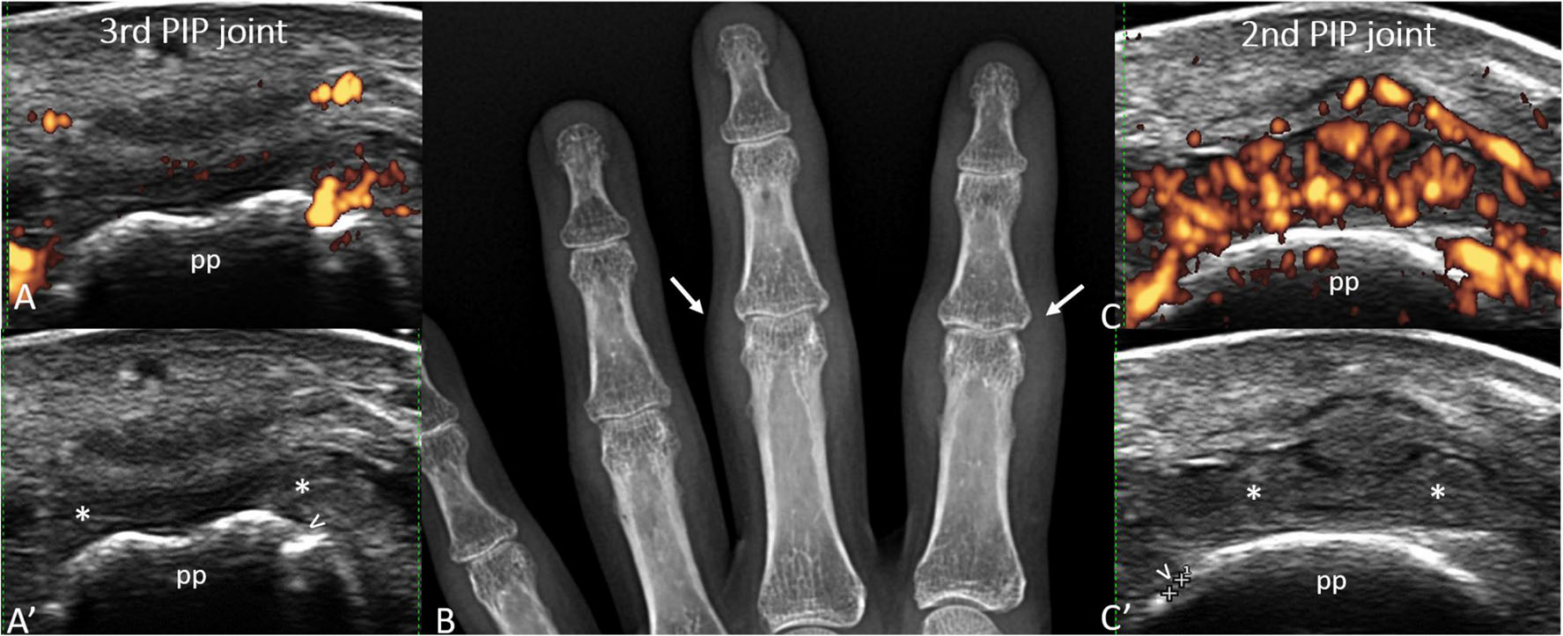
SLE-arthritis. Transverse ultrasound (US) scans of the dorsal aspect of 3^rd^ (A-A’) and 2^nd^ (C-C’) proximal interphalangeal joints, with (**A** and **C**) and without (**A**’ and **C**’) power Doppler mode, acquired using 10-22 MHz probe. US images show the presence of small bone erosions (arrowheads) and synovial hypertrophy (asterisks) with power Doppler signal (red spots). Soft tissue swelling (arrows), but no bone erosion, is visible on conventional radiography (**B**). Legend. Calipers: 0.48 mm bone erosion; pp = proximal phalanx

Although the clinical and prognostic relevance of bone erosions in patients with SLE has been poorly investigated, their detection and recognition appear to be relevant, especially in SLE patients with clinical and/or US findings of ‘active’ joint inflammation. Intuitively, the presence of bone erosions delineates a more aggressive phenotype of SLE-arthropathy. Therefore, the early identification of bone erosions may raise important implications for the management (including the therapeutic approach) of SLE patients with ‘active’ arthritis; in these patients, treatment should be aimed at preventing further joint damage and disability. In RA, bone erosions are important prognostic biomarkers for disease severity; their presence has been associated with poor functional outcome and irreversible loss of function [43–45]. In this context, sensitive imaging tools, such as US and MRI, have the potential to provide valuable information regarding the detection of joint damage in SLE patients; the value of these imaging tools is arguably highest in patients with very early inflammatory arthritis, where the sensitivity of CR (the reference imaging tool for the assessment of joint damage in RA) has been proven to be low (Fig. 1).

### Tendons and Entheses

**Fig. 2.**
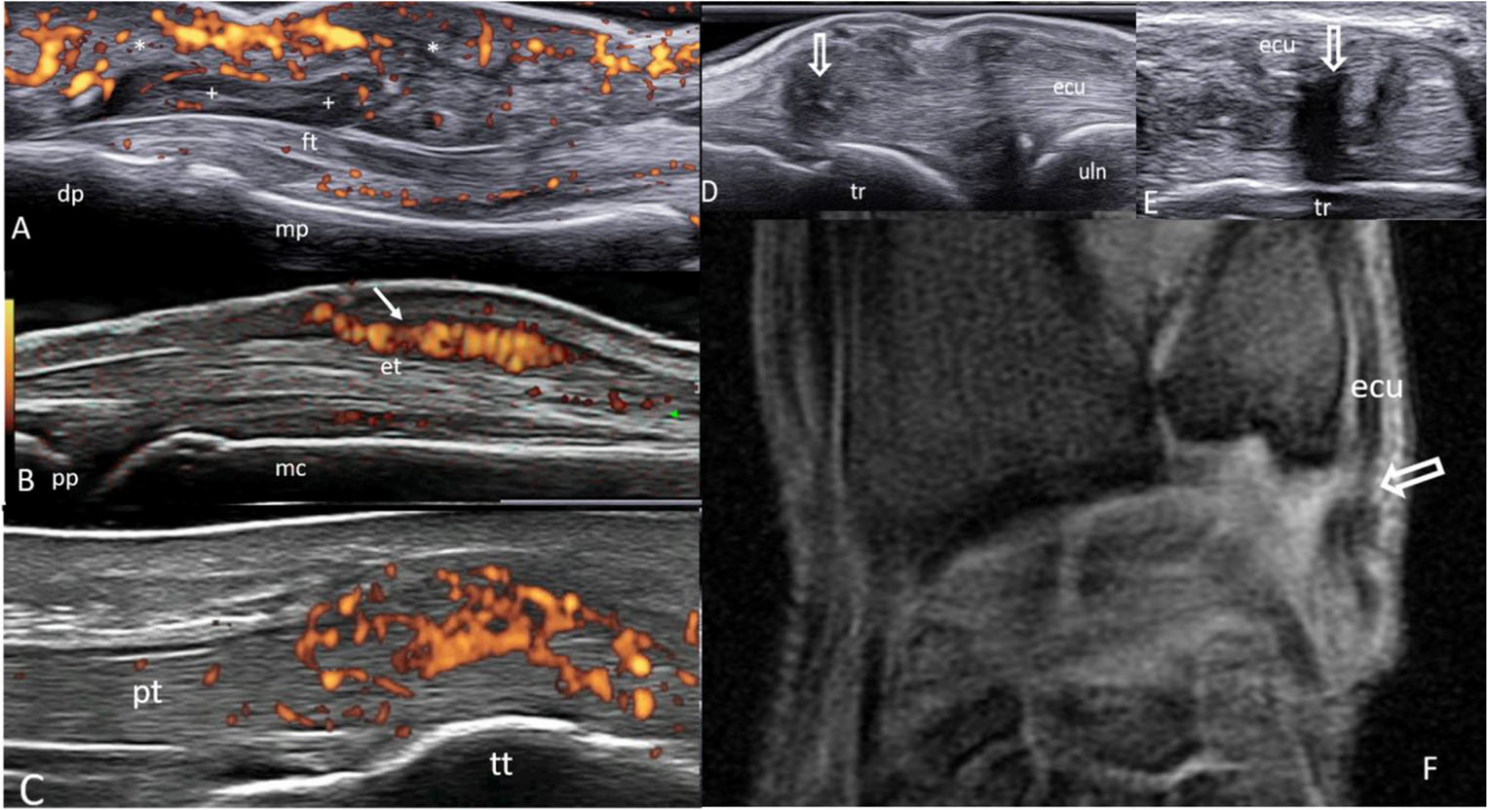
Tendon involvement in SLE. **A** Longitudinal volar ultrasound (US) scan of a 3rd finger of the hand obtained with a 8–24 MHz probe showing ‘active’ tenosynovitis (synovial hypertrophy within the tendon sheath: +) of the flexor digitorum profundus tendon. Note the presence of marked soft tissue edema (asterisks). **B** Longitudinal dorsal US scan of a 3rd metacarpophalangeal joint obtained with a 6–18 MHz probe showing peritendinitis (arrow) of the finger extensor tendon. **C** Longitudinal US scan of the distal insertion of a patellar tendon obtained with a 6–18 MHz probe depicting ‘active’ enthesitis, characterized by entheseal thickening and intense power Doppler signal (red spots). Intra-tendinous tear (open arrow) of an extensor carpi ulnaris tendon by US (**D**, longitudinal scan; **E**, transverse scan) and corresponding magnetic resonance imaging (**F**). dp = distal phalanx; ecu = extensor carpi ulnaris; et = finger extensor tendon; ft = flexor digitorum profondus tendon; mc = metacarpal bone; mp = middle phalanx; pp = proximal phalanx; pt = patellar tendon; tr = triquetrum. tt = tibial tuberosity; uln = ulnar styloid

In recent years, several imaging studies, mainly of US, have demonstrated a high prevalence of tendon pathology, indicating inflammation (i.e., tenosynovitis, tendinitis, and peri-tendinitis), and/or structural damage (i.e., tendon thinning, tendon dislocation, and tendon tear) in patients with SLE (Fig. 2).

In 2018, our research group detected a wide spectrum of tendon abnormalities in a cohort of 25 SLE patients with current or previous joint and/or tendon pain [46]. US abnormalities were found in 70 out of the 215 (32.6%) scanned tendons. Tenosynovitis was the most observed US abnormality (17.7% of the scanned tendons), followed by tendon dislocation (8.4%), tendinitis/peritendinitis (4.7%), and tendon thinning (4.2%). US findings indicating tendon tear were described at the extensor carpi ulnaris tendon in one patient. In a study by Delle Sedie et al. tenosynovitis was documented in 14 out of 50 (28.0%) SLE patients; tendon inflammation was detected at the hands (i.e., 2nd and 3rd finger flexor tendons) in 8 out of 14 (57.2%) patients, at the wrists (i.e., extensor carpi ulnaris tendon) in 3 (21.4%) patients, and at both the hands and wrists in the remaining 3 (21.4%) patients [47]. The prevalence of tenosynovitis was even higher (65%) in a study out by Wright et al. which was carried out on 17 SLE patients with hand arthritis [48]. Zollars et al. investigated the spectrum of MRI pathological findings in the hands and wrists of 20 SLE patients with hand arthritis; tenosynovitis was found in 12 out of 20 (60%) patients, most frequently at the tendons of the wrist [49].

Tendon rupture is a severe, albeit rare, manifestation of SLE. In a recently published case report, El Ouazi et al. showed the value of US and MRI in the diagnosis of bilateral patellar tendon rupture in a patient with SLE [50]. Hosokawa et al. highlighted the case and the imaging findings of a SLE patient with spontaneous rupture of the flexor tendon of the little finger [51].

SLE-arthropathy has been traditionally regarded as a process which involves synovial structures. Interestingly, recent studies have shown that also nonsynovial structures, such as the entheses and tendons without synovial sheath, may represent potential targets of SLE [6, 7]. Our research group explored the prevalence and distribution of US entheseal changes at the lower limb entheses of 65 patients with SLE, taking as controls 50 patients with psoriatic arthritis and 50 healthy subjects [7]. In this study, one or more US abnormalities were found in at least one enthesis in 44 out of 65 SLE patients (67.7%). US ‘active’ inflammation at the enthesis was more prevalent in SLE patients than in healthy subjects (67.7% vs 44.0%, *p*<0.001). The tibial insertion of the patellar tendon was the most involved enthesis, followed by the calcaneal insertion of the Achilles tendon. Of note, the presence of PD signal at the enthesis was associated with higher SLE disease activity scores (SLEDAI-2k *p*<0.001, β=0.52; MSK-BILAG *p*<0.001, β=0.56). Indeed, the detection of US inflammation at the enthesis opens a window to a previously unexplored area of research in SLE. Whether the presence of US enthesitis may identify a distinct subset of SLE arthropathy, or represent an imaging biomarker of increased risk of tendon rupture, are unanswered questions which require further investigations.

## Muscle Involvement in SLE

### Myositis

Diffuse myalgia and muscle tenderness are observed in up to 80% of SLE patients, more commonly during disease flares [52]. Inflammatory myositis [often with increased creatin-phosphokinase (CPK) levels] has an incidence of 4%–16% in cohorts from western countries [53, 54]. In a study by Garton et al., patients with SLE and myositis and patients with idiopathic inflammatory myopathies (IIMs) were followed-up for 20 years; no substantial difference in terms of morbidity or mortality was found between these two groups [55]. Dayal et al. compared the clinical and laboratory features of patients with SLE and myositis with patients with SLE without inflammatory muscle disease [53]. Patients with SLE and associated myositis were more likely to have alopecia (50% vs 17.6%, *p*=0.02), oral ulcers (80% vs 28.9%, *p*=0.001), erosive joint disease (60% vs 5%, *p*<0.001), and anti-RNP antibodies (80% vs 21%, *p*<0.001) than SLE patients without myositis. In a study by Cotton et al., non-Caucasian ethnicity, arthritis, Raynaud’s phenomenon and anti-Smith antibodies were the variables most significantly associated with a higher risk of developing myositis in a large cohort of SLE patients [56].

MRI represents the imaging gold standard in patients with suspected inflammatory myositis. Indeed, MRI accurately depicts the extent and severity of the muscle inflammatory abnormalities, and it is useful in guiding biopsy [57].

**Fig. 3.**
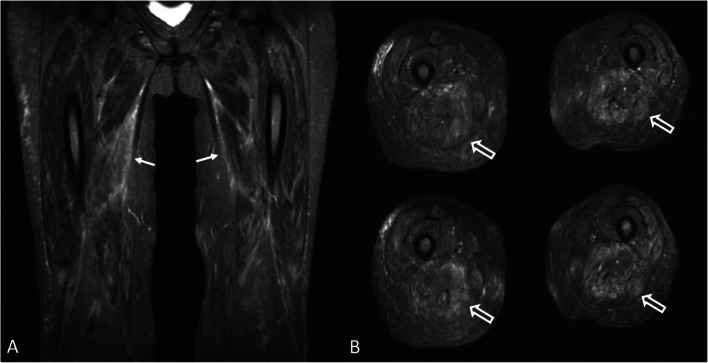
‘Active’ myositis in a SLE patient. Magnetic resonance imaging shows diffuse bilateral edema (arrows) within the adductors muscles on coronal STIR image **A**. Axial STIR images **B** show edema and inflammation of posterior thigh muscles (open arrow)

MRI findings of ‘active’ SLE myositis seem to not differ from those of IIMs; these are represented by high signal intensity of muscle belly (and decreased fat signal) on short tau inversion recovery (STIR) images (Fig. 3).

**Fig. 4.**
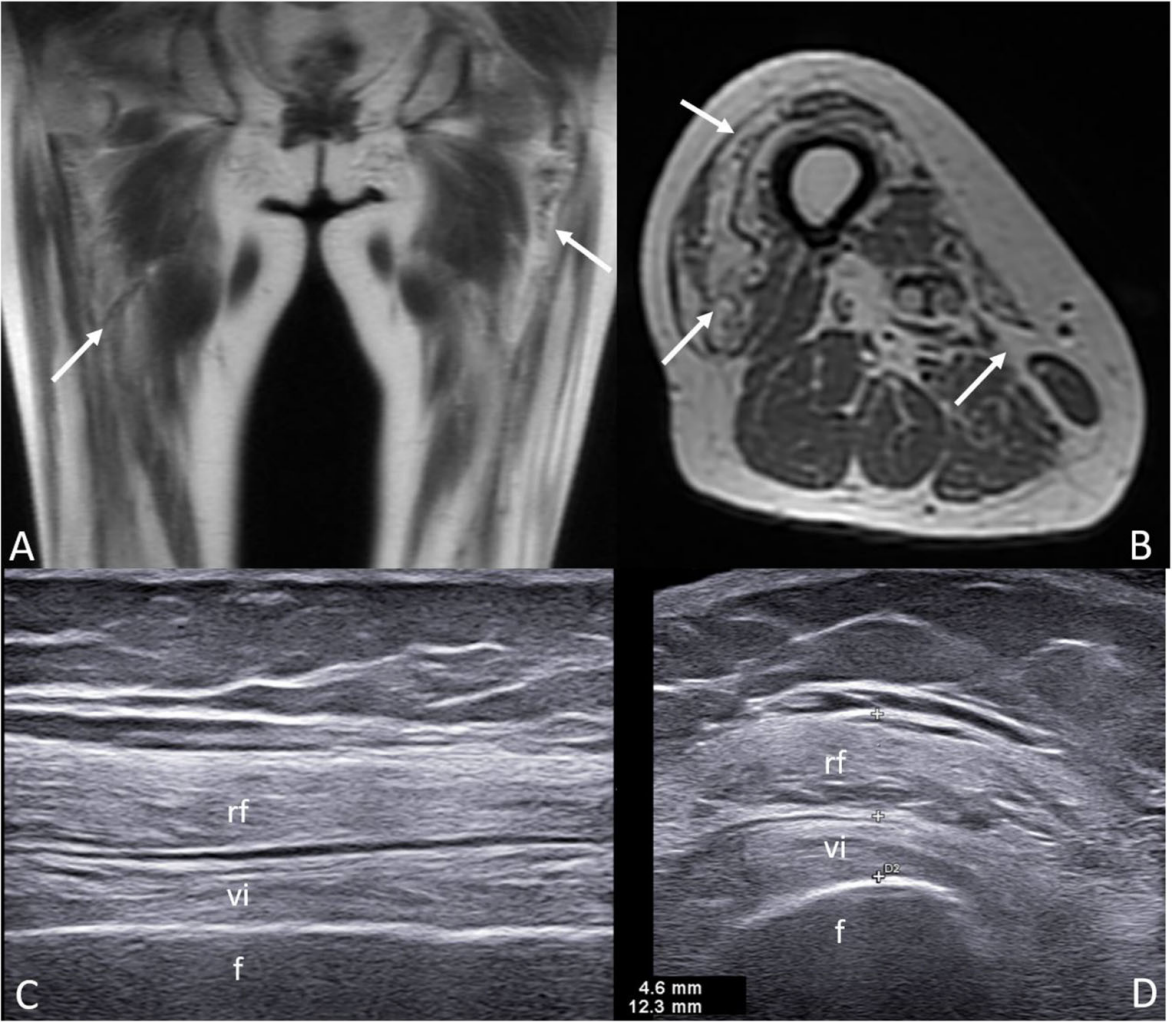
Chronic myositis in a SLE patient. Magnetic resonance imaging shows muscle reduction with advanced fatty infiltration (arrows) of quadriceps muscle bulk on unenhanced coronal (**A**) and axial (**B**) T1-weighted images. Ultrasound longitudinal (**C**) and transverse (**D**) scans obtained with a 3–11 MHz probe at the anterior thigh level show severely reduced thickness of rectus femoris (rf) and vastus intermedius (vi) muscles. The muscles bellies show a markedly increased echogenicity due to fatty replacement. f = femur

In the chronic phases, fatty replacement of the muscle is the dominant abnormality which can be best appreciated as bright signal on T1-weighted sequences (Fig. 4A and Fig. 4B).

T1-weighted sequences also provide excellent anatomic detail of various muscle groups and quantitative assessment of muscle atrophy. By depicting large areas of muscles, MRI facilitates the identification of the most abnormal site for biopsy, thus, increasing the diagnostic yield of this procedure [58].

US, including elastography, is emerging as a promising tool for the assessment of muscle pathology (i.e., inflammatory myositis) in rheumatic patients [59–61, 62••]. The main parameters for the US assessment of myositis are muscle thickness and muscle echogenicity. In the ‘early’ stages of myositis, muscle thickness is usually normal while a decrease of echogenicity, indicative of edema, could be observed [63]. In the later stages (Fig. 4C and Fig. 4D), a progressive reduction of muscle thickness and an increase of echogenicity (due to fibrous-adipose replacement) are the dominant US findings [64].

### Drug-Induced Myopathy

An inflammatory myositis must be differentiated from a drug-induced myopathy, a muscle pathological condition which has been mostly associated with antimalarials and steroid use (and potentially statins) in SLE patients [65].

In a case-control study, Tselios et al. found that chronic antimalarials use was a potential risk factor for elevation of muscle enzymes in SLE patients [66]. The prevalence of muscle enzymes abnormalities among antimalarials users was 16.3%, with myopathy and clinical weakness developing in about 2.5% of patients. Steroid-induced myopathy usually presents with insidious onset and slowly progressive painless muscle weakness, which usually involves the proximal muscles of the lower limbs and the pelvic girdle [67]. Muscle enzymes are usually normal or slightly elevated, and electrophysiology shows a myopathic pattern in the late stages. Steroid-induced myopathy is more likely to be caused by fluorinated glucocorticoids, such as dexamethasone, betamethasone, and triamcinolone, than by nonfluorinated glucocorticoids [68].

The role of imaging in the assessment of drug-induced myopathy has been poorly investigated. Hatakenaka et al. observed a prolongation of MRI T2 relaxation time in patients with steroid-induced muscle atrophy [69]. Peters et al. found an association between MRI muscle edema/lipomatosis, CPK levels, and muscle weakness in patients with lipid-lowering therapy–associated myopathy [70]. Recent US studies showed an increase of muscle echogenicity in steroid-induced myopathy, with or without reduction of muscle thickness [71, 72].

## Bone Involvement in SLE

### Osteonecrosis

Osteonecrosis (also called avascular necrosis, aseptic necrosis, or ischemic necrosis of bone) is a clinical syndrome characterized by death of subchondral bone, as the results of insufficient blood supply, which leads to trabecular and subchondral collapse with consequent pain, impairment, and permanent joint damage [73]. Osteonecrosis represents a serious comorbidity in patients with SLE. The prevalence of symptomatic osteonecrosis in SLE patients varies between 4% and 15%; interestingly, this goes up to 30% in asymptomatic SLE patients [74, 75]. In SLE, osteonecrosis is frequently multifocal, with up to 50% of patients showing involvement of multiple sites at the diagnosis. The hip joint and the knee joint (i.e., the femoral head and tibial plateau) are the most involved sites [73, 75].

Acute and severe joint pain in one or a few joints, most commonly the hip joint, may indicate the development of osteonecrosis, especially in patients on long-term corticosteroid therapy.

**Fig. 5.**
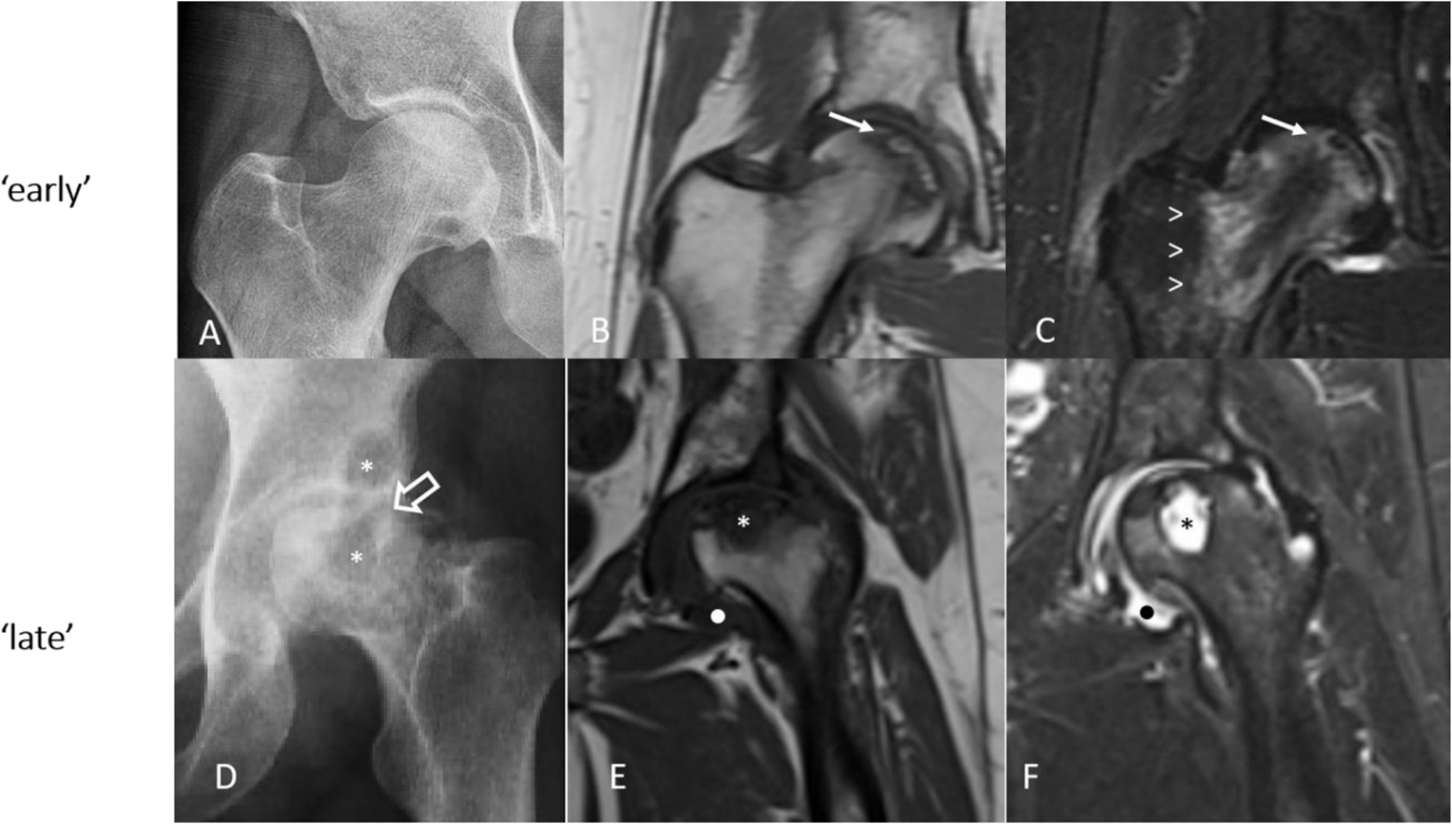
‘Early’ hip osteonecrosis in a SLE patient (**A-C**). Conventional radiography **A** shows mild and initial signs of osteoarthritis (i.e., subchondral sclerosis of the femoral head). Coronal magnetic resonance imaging (T1-weighted, **B**; STIR, **C**) shows the characteristic ‘double line’ sign (arrow) and the presence of diffuse bone marrow edema (arrowheads). ‘Late’ hip osteonecrosis in a SLE patient (**D-F**). Conventional radiography (**D**) shows osteochondral collapse (open arrow) and irregularity of the femoral head. There is cystic change within the femoral head and subchondral cyst formation within the lateral acetabulum (asterisks). Magnetic resonance imaging (T1-weighted, **E**; STIR, **F**) confirms the subchondral collapse in the weightbearing femoral head with a large area of subchondral damage. Note the presence of cystic change within the femoral head (asterisks) and secondary synovitis of the hip joint (rounded dots)

Imaging has a key diagnostic role [76]. MRI has a great utility in the early stages of osteonecrosis. Within epiphyseal lesions (i.e., osteonecrosis of the hip), MRI early changes include a central necrotic area with preserved fat-signal surrounded by an irregular sclerotic and reactive rim [76, 77]. This gives the typical ‘double line sign’, which is best seen on T2 weighted sequences (Fig. 5).

In advanced phase, normal bone is replaced by fibrotic tissue which appears as low intensity signal in both T1w and T2w sequences. Other unspecific MRI findings include bone marrow edema, synovial effusion, and secondary osteoarthritic changes [76, 77]. CR is typically insensitive for the detection of early changes of osteonecrosis; these usually require several months to occur (Fig. 5A). Patchy areas of subchondral radiolucency and sclerosis represent the earliest CR changes. Later, subchondral radiolucency can organize into the ‘crescent sign’, which is pathognomonic for osteonecrosis. In advanced stages, flattening or collapse of the articular surface, with loss of the normal bone morphology, bone fragmentation, joint space narrowing, and secondary osteoarthritis, can occur (Fig. 5D) [76, 77].

CT can be useful for the detection of subchondral fracture, for the identification of subtle head collapse that is not detected on MRI, as well as for the assessment of the severity of secondary osteoarthritis [77]. Bone scintigraphy detects osteoblastic activity and blood flow in the early phases of osteonecrosis, and it can be considered when MRI is unavailable/contraindicated, or in the clinical suspect of multifocal osteonecrosis [78].

### Osteomyelitis and Septic Arthritis

Infection is a major source of morbidity and mortality in SLE patients worldwide [79]. Bone and joint infections (respectively osteomyelitis and septic arthritis) are uncommon but potentially life-threating complications of SLE.

Osteomyelitis is more prevalent in SLE patients than in the general population, and in the former group it occurs at a younger age [80, 81]. In SLE patients, osteomyelitis often affects the bones of the lower limbs, in particular the tibia and the femur [80, 81].

Osteomyelitis should be suspected in the following clinical scenarios: (a) new or worsening MSK pain, particularly when this is associated with fever and/or bacteremia; (b) in patients with poorly healing wounds adjacent to bony structures (e.g., patients with chronic skin ulcers); (c) in patients with signs of soft tissue infection overlying previously implanted orthopedic hardware; (d) in patients with traumatic injury and (e) in patients with septic arthritis.

Imaging has a major role in the early assessment and recognition of bone infection [82–85].

**Fig. 6.**
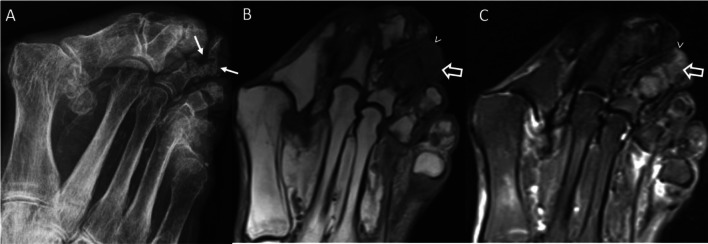
Osteomyelitis of the third toe in a SLE patient. Conventional radiography **A** shows diffuse bone destruction of middle and distal phalanges of the third toe (arrows). Magnetic resonance imaging shows diffuse low T1-weighted signal (**B**; open arrow) and increased STIR signal (**C**; open arrow) within the medullary cavity of the intermediate and terminal phalanges of the third toe, which is associated with marked soft tissue swelling (arrowheads)

MRI should be regarded as the first-line imaging tool in patients with early disease (<2 weeks after bone infection). MRI depicts bone marrow edema, joint and soft tissue inflammation, and provides information on the extent of cortical bone destruction (Fig. 6) [82–85]. The use of gadolinium improves the distinction between abscess, phlegmon, and necrosis.

CR findings may be absent or nonspecific in the first 2 weeks following bone infection. In patients with a relatively long disease duration (>2 weeks), CR may detect soft tissue swelling, osteopenia, cortical loss, bony destruction, and periosteal reaction (Fig. 6). In chronic osteomyelitis (≥6 weeks after bone infection), reactive sclerosis, sequestrum (i.e., segment of necrotic bone that becomes separated from the healthy adjacent bone), and involucrum (i.e., a thick sheath of periosteal new bone surrounding a sequestrum) may occur [83, 84].

CT and nuclear studies (e.g., tagged-white blood cell scan) are valuable alternatives to MRI when this is contraindicated, or in presence of metallic hardware which may impair the diagnostic accuracy of MRI [86, 87]. CT is more sensitive than CR for the assessment of cortical and trabecular integrity and periosteal reaction, it can depict sequestrum, which appears as a sclerotic lesion with a lucent rim, and involucrum formation. On bone scintigraphy scan with technetium-99m-methylene diphosphonate, osteomyelitis appears as focal hyperperfusion, focal hyperemia, and focally increased bone uptake [88]. Pre-existing bone pathologies, such as degenerative joint disease or fracture, may also generate increased bone uptake and therefore limit the specificity of this imaging method. In these circumstances, white blood cell scans may improve the specificity of the nuclear imaging findings, despite having a limited ability in distinguishing between osteomyelitis and soft tissue infections [89]. Gallium scans are useful to provide information about the ‘activity’ status of the osteomyelitic lesions.

Septic arthritis is a medical emergency which is associated with severe morbidity and high mortality risk. In SLE patients, it is usually oligoarticular and frequently involves the hip joint [90]. Predisposing factors for septic arthritis in SLE include systemic and local corticosteroids, ‘active’ disease, osteonecrosis, and joint synovitis.

Imaging is useful to evaluate the presence and the extent of the infectious process, to assess the integrity of the articular structures, and to help guiding the needle for diagnostic joint aspiration [91].

MRI changes appear very early in the course of the disease (as soon as 24 h after the onset of infection). MRI can reveal findings suggestive of infection at the bone (e.g., bone erosions and hyaline cartilage destruction) and/or at the soft tissues (i.e., synovial/capsular enhancement, peri-synovial edema, and joint effusion) [92].

CR can be normal in the early stages of septic arthritis or may reveal nonspecific findings (i.e., periarticular osteopenia and/or soft tissues swelling). In the late stages, periosteal reaction, bone destruction, and sequestrum formation often occur. US can detect articular (i.e., joint effusion) and/or periarticular effusion (i.e., bursal fluid collections, soft-tissue abscesses) and it is useful to guide the direction of the needle for diagnostic aspiration [93, 94]. CT shows soft tissue swelling and joint space widening in the early stages; joint space narrowing, increased density of fatty marrow, periosteal reaction, gross bone erosions, and joint destruction can be observed in the late stages of the disease.

### Osteoporosis and Fragility Fractures

Osteoporosis and fragility fractures are well-known comorbidities in SLE patients [95]. Glucocorticoid use, systemic inflammation, reduced mobility (i.e., sedentary lifestyle), and vitamin D deficiency are traditional risk factors for osteoporosis which have been commonly reported in SLE patients [96].

Several studies have demonstrated that osteopenia and osteoporosis are more prevalent in SLE patients than in matched controls [97, 98]. Moreover, in a recent study by Tedeschi et al., SLE patients showed a 2-fold higher fracture risk in comparison with matched comparators (HR 2.09 [95% CI 1.85–2.37]; *p*<0.01) [99].

Universally, DEXA is the mainstay for the diagnosis and monitoring of bone status. DEXA values of bone mineral density (BMD) correlate well with the bone strength and are used to define cut-offs for osteoporosis [100]. Nevertheless, fragility fractures (the major clinical manifestation of osteoporosis) can occur in up to 30% of SLE patients despite having a normal BMD [98, 101]. Moreover, it has been demonstrated that up to 20% of SLE patients may develop asymptomatic vertebral fractures and these, therefore, may go under-recognized [102].

CR is the first-line imaging tool for the detection of fragility fractures, especially at the spine. Other imaging techniques, namely, CT and MRI, could be used in certain clinical scenarios, such as: (a) for the detection of sacral fractures and/or occult stress fractures of the proximal femur; (b) for differentiating between acute and long-standing vertebral fractures; (c) for excluding neurological complications, such as spinal cord compression [103].

## Conclusions

With this review, we highlight the main imaging findings detectable in SLE patients with MSK involvement. US should be regarded as the first-line imaging tool for the assessment of inflammation and early structural damage in SLE patients with joint symptoms. MRI is extremely useful for the evaluation of muscle pathology (i.e., myositis) and for the early diagnosis of osteonecrosis. CR remains the traditional gold standard for the assessment of structural damage (i.e., bone erosions) in patients with SLE-arthritis. The diagnostic value of CR is affected by the low sensitivity in depicting early pathological changes. CT allows a detailed evaluation of bone damage. However, the inability to distinguish soft tissues and the need for ionizing radiation limit its use to selected clinical circumstances. Nuclear imaging has an important role in the evaluation of bone infection, especially MRI when is contraindicated. Finally, DEXA represents the imaging mainstay for the assessment of bone status in individuals with or at-risk of osteoporosis.
